# The Impacts of Nitrogen Pollution and Urbanization on the Carbon Dioxide Emission from Sewage-Draining River Networks

**DOI:** 10.3390/ijerph191610296

**Published:** 2022-08-18

**Authors:** Yongmei Hou, Xiaolong Liu, Guilin Han, Li Bai, Jun Li, Yusi Wang

**Affiliations:** 1 Tianjin Key Laboratory of Water Resources and Environment, Tianjin Normal University, Tianjin 300387, China; 2 School of Geography and Environmental Science, Tianjin Normal University, Tianjin 300387, China; 3 Institute of Earth Sciences, China University of Geosciences (Beijing), Beijing 100083, China

**Keywords:** urbanization, CO_2_ emission, nitrogen pollution, river networks, carbon cycling

## Abstract

Carbon dioxide (CO_2_) emissions from river water have sparked worldwide concerns due to supersaturate CO_2_ levels in the majority of global rivers, while the knowledge on the associations among nitrogen pollution, urbanization, and CO_2_ emissions is still limited. In this study, the CO_2_ partial pressure (*p*CO_2_), carbon and nitrogen species, and water parameters in sewage-draining river networks were investigated. Extremely high *p*CO_2_ levels were observed in sewage and drainage river waters, such as Longfeng River, Beijing-drainage River, and Beitang-drainage River, which were approximately 4 times higher than the averaged *p*CO_2_ in worldwide rivers. Correlations of carbon/nitrogen species and *p*CO_2_ indicated that carbon dioxide in rural rivers and sewage waters primarily originated from soil aeration zones and biological processes of organic carbon/nitrogen input from drainage waters, while that in urban rivers and lakes was mainly dominated by organic matter degradation and biological respiration. Enhanced internal primary productivity played critical roles in absorbing CO_2_ by photosynthesis in some unsaturated *p*CO_2_ sampling sites. Additionally, higher *p*CO_2_ levels have been observed with higher NH_4_^+^-N and lower DO. CO_2_ fluxes in sewage waters exhibited extremely high levels compared with those of natural rivers. The results could provide implications for assessing CO_2_ emissions in diverse waters and fulfilling water management polices when considering water contamination under intense anthropogenic activities.

## 1. Introduction

River ecosystems play dominant roles in conveying materials between inland water and oceans, which has implications for the circulation of matter and energy in the global carbon pool, as well as the global carbon budget and climate change [1,2]. Generally, the supersaturate carbon dioxide (CO_2_) levels of most river ecosystems cause CO_2_ to be released from rivers to the atmosphere, making river ecosystems a vital source of the total global CO_2_ emissions [3,4]. Global CO_2_ emissions have been estimated at 1.80 petagrams of carbon per year (Pg C·yr^–1^) from rivers and 0.32 Pg C·yr^–1^ from lakes or reservoirs, being equivalent to approximately 2.34 times the total carbon transferred from inland water to oceans (0.90 Pg C·yr^–1^) [5,6]. Owing to the enormous challenges of carbon assessment and environmental management in the global ecosystem, CO_2_ emissions from river networks have inspired great interest around the world. Therefore, much efforts have been made to assess the contributions of CO_2_ emissions from natural river ecosystems, e.g., Amazon and Mekong River systems [7], Guadalete River [8], and the Wujiang River [9]. However, urban river networks are intensively impacted by anthropogenic activities (e.g., sewage draining and urban land coverages) compared with those of natural river networks, which generally exhibit more complex watershed environmental conditions dominating the production and transportation of CO_2_ [10]. Integrated studies have affirmed that watershed urbanization profoundly amplifies the uncertainty of riverine CO_2_ emissions, while the fact that few works have concentrated on urban river networks suggests more efforts to fill the gap [11].

Mechanisms of CO_2_ emissions from river ecosystems are associated with a combination of internal factors (temperature, pH, dissolved oxygen, and organic matters) and external factors (geomorphology, hydrology, precipitation, and anthropogenic activities) [8,12], which are further impacted by autotrophic and heterotrophic microbial activities in waters, photosynthesis of aquatic vegetation, organic matter degradation, and mineralization [13]. The drastic disturbance in urban river networks commonly expressing low dissolved oxygen (DO) and high nutrient or organic carbon concentration is closely related to the higher partial pressure of carbon dioxide (*p*CO_2_) levels than rivers in less impacted areas. It is principally attributed to the fact that nutrients stimulate microbe reproduction and modify biochemical environmental conditions, accelerating CO_2_ release from waters [14,15]. Multiple studies have also emphasized that the significantly different nutrient/carbon inputs from draining waters and soil CO_2_ influxes between various urban rivers would profoundly enlarge the uncertainty of CO_2_ emissions from regional river ecosystems [16,17,18]. Although these works supplied some valid results, the correlations between anthropogenic nutrient loading and CO_2_ emissions, and the factors affecting on the distributions of CO_2_ emissions from urban rivers, are still limited. The lack of investigation on overall river networks in specific regions, which has primarily concentrated on local rivers, lakes, and estuaries [19,20,21], is also not conducive to the construction of a global carbon budget system.

Enormous regions around the world have been experiencing rapid urbanizations. The uncertainty and complexity of sources of contaminant waters (especially domestic/industrial sewage-draining water and runoff coming from agricultural soil) would destroy the nitrogenous and carbonic balance of aquatic ecosystems [18]. Hence, focusing on CO_2_ emissions in urban river networks is beneficial to recognize the response of aquatic ecosystems to anthropogenic activities as well as assessing the global carbon budget. Tianjin, one of the most prosperous coastal cities in China, receives sewage waters from local and surrounding areas (including Beijing and Hebei Province), regarded as important sources of CO_2_ emissions. The aims of this study are to (1) disclose the spatial distribution patterns of *p*CO_2_ and CO_2_ emission fluxes from different waters; (2) explore the mechanisms producing CO_2_ with various nitrogen concentrations; and (3) reveal the primary factors affecting CO_2_ emissions in a eutrophic aquatic environment. The results of this study would be helpful for assessing the source and sink effects of CO_2_ in urban and rural rivers under the influence of multiple factors, providing a database for future studies on rivers with similar characteristics of nitrogen pollution and urbanization worldwide, and fulfilling water management polices when considering the contribution of global urban rivers to carbon cycling and CO_2_ emissions.

## 2. Materials and Methods

### 2.1. Study Site

**Figure 1:**
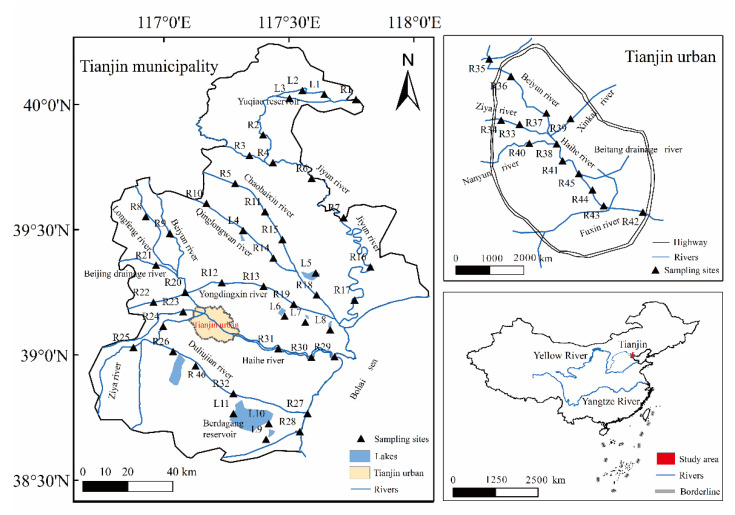
Map of sampling sites in Tianjin river network.

Tianjin (38°34′–40°15′ N, 116°43′–118°04′ E) is a densely populated (13.87 million people) coastal metropolis in China, located northeast of North China Plain and downstream of the Haihe River Basin, which is enclosed to the east by the Bohai Sea. The climate in the area is warm temperate sub-humid monsoon, with higher temperature in the summer (monthly average temperature of 28 °C) and an average annual temperature of 14.8 °C. The mean annual precipitation is 520–660 mm with approximately 75% occurring from June to August. There are five major rivers, the Haihe River, Yongdingxin River, Chaobaixin River, Duliujian River, and Jiyun River, in Tianjin (Figure 1). Among these, the Haihe River flows through the urban area and eventually enters into the Bohai Sea. Tianjin serves as one of the most important economic centers of China experiencing rapid urbanization processes, which convert the river ecosystem due to anthropogenic activities. Tianjin river networks accept sewage from the neighboring areas as well as load their own water environmental pollution, threatening the water resource safety, to which more attention should be paid [22].

### 2.2. Sampling and Analysis

Previous studies have verified that summer is a sensitive period for CO_2_ production and emissions because of promoted and active carbon biogeochemical processes [10,23]. Based on the climatic conditions and characteristics of the main rivers, water samples were collected throughout the Tianjin municipality in June 2015, including 11 sampling sites in lakes/reservoirs, 5 sampling sites in sewage-draining waters, and 41 sampling sites in rivers (Figure 1). Among them, 18 samples in urban rivers (mainly in Haihe River) and 23 sampling sites in rural rivers were deployed to better investigate the CO_2_ emissions under various anthropogenic activities.

Surface water samples (depth 0.5 m) were collected by a Niskin sampler in triplicate and all the water samples were stored away from light at 4 °C until analysis in a laboratory. The water physical–chemical variables, including water temperature (T), dissolved oxygen (DO), electric conductivity (EC), pH, and total dissolved solids (TDSs), were determined in situ with a multi-parameter monitor (Yellow Springs Instrument, YSI-6600 V2, United States Gimcheon Instruments Inc.). Alkalinity (Alk) of samples was measured using a titration method with 0.01 mol·L^−1^ HCl in the field. The concentration of dissolved inorganic carbon (DIC) in water was calculated by the carbonate equilibrium system, detailed formulations are shown in Section 2.3. The concentration of dissolved organic carbon (DOC) in water was measured with an elemental analyzer (Vario TOC cube, Elementar, Langenselbold, Germany). The concentrations of NH_4_^+^-N, NO_3_^−^-N, NO_2_^−^-N, and total nitrogen (TN) were determined using automatic flow analysis (AA3 Auto Analyzer, SEAL, Norderstedt, Germany) in a laboratory. However, because high concentrations of organic matter and heavy metals in sewage water and effluents would affect the accuracies of the measurements of TN, the AA3 Auto Analyzer was equipped with Cd columns and effective UV lamps to ensure the digestion rate. By following the protocols of the People’s Republic of China State Environmental Protection Standards: Determination of Total Nitrogen by Continuous Flow Analysis (CFA) and N- (1-naphthyl) Ethylene Diamine Dihydrochloride Spectrophotometry (Chinese Edition) (HJ 667-2013), the concentrations of total nitrogen were determined. Dissolved organic nitrogen (DON) was calculated by subtracting the dissolved inorganic nitrogen (DIN, including NH_4_^+^-N, NO_3_^−^-N, and NO_2_^−^-N) from TN. The authoritative protocol manual handbook in China (The water and wastewater monitoring and analysis methods (version 4)) was applied to determine the sewage-draining waters. Laboratory standards, blanks, and replicates were applied to guarantee that the accuracies of all the analyses were better than ±5%.

### 2.3. The Calculation of Partial Pressure of Carbon Dioxide in River Waters

The principal buffer mechanism in a freshwater system is carbonate equilibrium. H_2_CO_3_, HCO_3_^−^, CO_3_^2−^, and aqueous CO_2_ make up the total dissolved inorganic carbon (DIC) in water, which is affected by pH, water temperature, and ionic strength [24,25]. Previous studies have reported that the alkalinity–pH–temperature method might overestimate the values of *p*CO_2_ at low pH, while they could be accurately described with pH > 7.2 and total alkalinity > 1 mmol·L^−1^ [25,26]. Based on the pH (ranging from 7.29 to 10.41) and alkalinity (exceeding 1.43 mmol·L^−1^) in water samples in this study area, the alkalinity–pH–temperature method is employed to calculate *p*CO_2_. The *p*CO_2_ values are calculated according to the carbonate equilibrium system which is described by the following equations:

(1) $${\mathrm{CO}}_{2}+H_{2}O↔H_{2}{\mathrm{CO}}_{3}^{*}↔H^{+}+{\mathrm{HCO}}_{3}^{-}↔2H^{+}+{\mathrm{CO}}_{3}^{2-},$$

(2) $$K_{{\mathrm{CO}}_{2}}=\frac{\left[H_{2}{\mathrm{CO}}_{3}^{*}\right]}{{p\mathrm{CO}}_{2}}=10^{\left(7\times 10^{-5}\times T^{2}-0.016T-1.11\right)},$$

(3) $$K_{1}=\frac{\left[H^{+}]\times [{\mathrm{HCO}}_{3}^{-}\right]}{\left[H_{2}{\mathrm{CO}}_{3}^{*}\right]}=10^{\left(-1.1\times 10^{-4}\times T^{2}+0.012T-6.58\right)},$$

(4) $$K_{2}=\frac{\left[H^{+}]\times [{\mathrm{CO}}_{3}^{2-}\right]}{\left[{\mathrm{HCO}}_{3}^{-}\right]}=10^{\left(-9\times 10^{-5}\times T^{2}+0.0137T-10.62\right)},$$

where K_i_ are DIC dissociation constants related to temperature. Based on these, *p*CO_2_ (μatm), H_2_CO_3_*, and CO_3_^2−^ are evaluated with HCO_3_^−^, water temperature (T), and pH, and the relative calculating equations are as follows.

(5) $$p{\mathrm{CO}}_{2}=\frac{\left[H_{2}{\mathrm{CO}}_{3}^{*}\right]}{K_{{\mathrm{CO}}_{2}}}=\frac{\alpha \left(H^{+}\right)\times \alpha \left({\mathrm{HCO}}_{3}^{-}\right)}{K_{{\mathrm{CO}}_{2}}\times K_{1}}$$

(6) $$\alpha \left(H^{+}\right)=10^{-\mathrm{pH}}$$

(7) $$\alpha \left({\mathrm{HCO}}_{3}^{-}\right)=\left[{\mathrm{HCO}}_{3}^{-}\right]$$

(8) $$\left[H_{2}{\mathrm{CO}}_{3}^{*}\right]=\frac{10^{-\mathrm{pH}}\times \left[{\mathrm{HCO}}_{3}^{-}\right]}{K_{1}}$$

(9) $$\left[{\mathrm{CO}}_{3}^{2-}\right]=\frac{K_{2}\times \left[{\mathrm{HCO}}_{3}^{-}\right]}{10^{-\mathrm{pH}}}$$

### 2.4. CO_2_ Emission Flux Calculation

CO_2_ concentration gradient and gas exchange coefficient commonly govern the CO_2_ exchange rate between water and atmosphere [2]. According to the theoretical diffusion model of CO_2_, which has been efficiently employed at the water–air interface in previous studies [5,14], the CO_2_ emission fluxes in this work are evaluated as follows:

(10) $$F_{{\mathrm{CO}}_{2}}=K_{{\mathrm{CO}}_{2}}\times k\times \left(p{\mathrm{CO}}_{2.\mathrm{water}}-p{\mathrm{CO}}_{2.\mathrm{air}}\right),$$

where $F_{{\mathrm{CO}}_{2}}$ (mmol·m^−2^·d^−1^) is the CO_2_ emission flux, $K_{{\mathrm{CO}}_{2}}$ (μatm) is the Henry’s law constant of CO_2_ calibrated by measured T (°C) in situ, k (cm·h^−1^) is the gas exchange coefficient at the air–water interface, $p{\mathrm{CO}}_{2.\mathrm{water}}$ and $p{\mathrm{CO}}_{2.\mathrm{air}}$ are the partial pressure of CO_2_ in water and air, respectively.

Among these, k is uncertainty that should be cautiously confirmed by specific environmental conditions. Researchers have made great efforts on combining multiple factors (such as the wind speed, temperature, stream slope, discharge, and depth) with estimation models that have been widely employed in previous studies to optimize the calculation model of gas transfer velocity [25,27,28]. Wind speed is a primary cause of turbulence in low-gradient river systems that strongly relate to k_600_ [7,29]. In addition, it was proposed that averaged k could be used to estimate gas fluxes from water [30]. Considering the low terrain and steady river flow velocity in this work, k is evaluated by a typical model primarily correlating it with the wind speed and temperature. We also adopted the averaged value (k = 4 cm·h^−1^) to calculate CO_2_ emission fluxes and inconspicuous differences between the two estimation methods were found. Relevant calculation equations are listed as follows [31]:

(11) $$k=k_{600}\times {\left(\frac{S_{C}}{600}\right)}^{x},$$

(12) $$k_{600}=2.07+0.215\times U_{10}^{1.7}.$$

(13) $$S_{C}=1911.1-118.11\times T+3.4527\times T^{2}-0.04132\times T^{3},$$

in which k_600_ is the gas exchange coefficient of CO_2_ calibrated with wind speed U_10_ (m·s^−1^) which is the wind speed 10 m above the water surface. The value of x is determined by U_10_, x = −0.67 or x = −0.5 when U_10_ < 3.7 m·s^−1^ and U_10_ > 3.7 m·s^−1^, respectively. S_C_ (cm·h^−1^) is the Schmidt number represented by T (°C). The monthly averaged wind speed of Tianjin in June (from 2014 to 2021) was obtained from the China Meteorological Data Network.

### 2.5. Statistical Analysis

IBM SPSS Statistics 19 was used for the descriptive statistical analysis, variance analysis, and linear correlation analysis. One-way analysis of variance (ANOVA) was performed to examine the differences in $p{\mathrm{CO}}_{2}$/$F_{{\mathrm{CO}}_{2}}$ from different sampling sites. Correlation coefficients between $p{\mathrm{CO}}_{2}$/$F_{{\mathrm{CO}}_{2}}$ and environmental parameters were determined by linear regression analysis. Statistical significance was found when the *p* value < 0.05 for all the variance analyses and linear correlation analyses. All the figures were made by Origin 2018, R Studio, and Grapher 15.

## 3. Results

### 3.1. Variations in Water Quality Parameters

Statistics of water quality parameters including water temperature (T), dissolved oxygen (DO), total dissolved solids (TDSs), electric conductivity (EC), pH, and alkalinity (Alk) are listed in Table 1. In June, surface water temperatures ranged between 25.60 °C and 34.80 °C. The pH ranged from 7.29 to 10.41 and the maximum value was detected in rural rivers (R23, Ziya River) while the minimum value was found in sewage-draining waters (R9, Longfeng-drainage River) (Figure 1). Large ranges of TDSs in river waters in Tianjin have been observed, with the highest value in the estuary of the Duliujian River (R27, 22,900 mg·L^−1^) and with lowest value in the Yuqiao Reservoir (L1, 250 mg·L^−1^). DO generally demonstrated oversaturated conditions in these waters, with an average value of saturation of 102%, and ranged from 2.15 to 14.24 mg·L^−1^.

**Figure 2:**
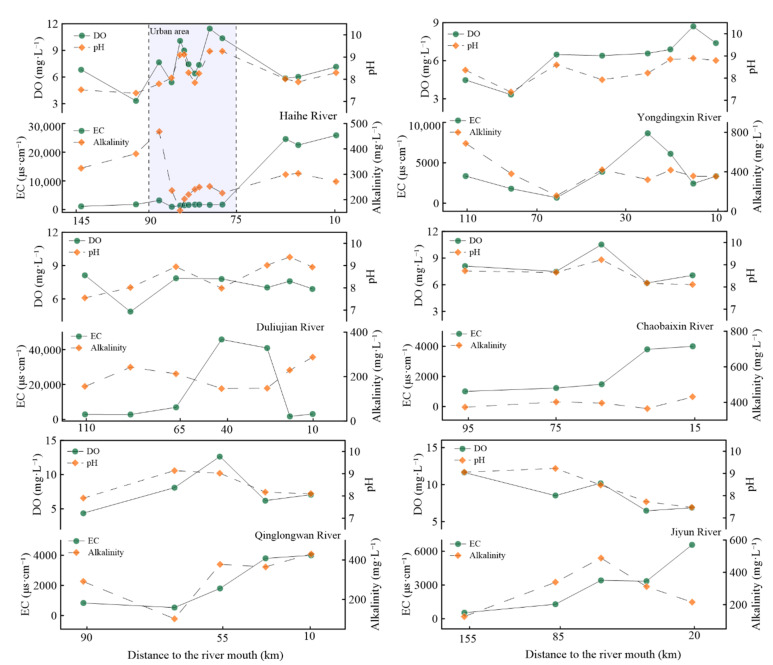
Longitudinal variations in DO, pH, EC, and Alk along major rivers in Tianjin.

As shown in Figure 2, from upstream to downstream, water parameters presented obvious longitudinal variations in all the studied rivers, with different spatial patterns. Since significant correlations (R = 0.79, *p* < 0.01) have been observed between DO and pH, the spatial distributions of DO and pH exhibited a similar tendency in all major rivers. In Haihe River, DO and pH increased remarkably in the urban area, while the EC and Alk decreased significantly in the same reaches. Except DO and pH in Jiyun River and Chaobaixin River, water parameters in most of the rivers increased from upstream to downstream and estuaries or coastal area.

### 3.2. Variations in Nitrogen and Carbon Species

**Figure 3:**
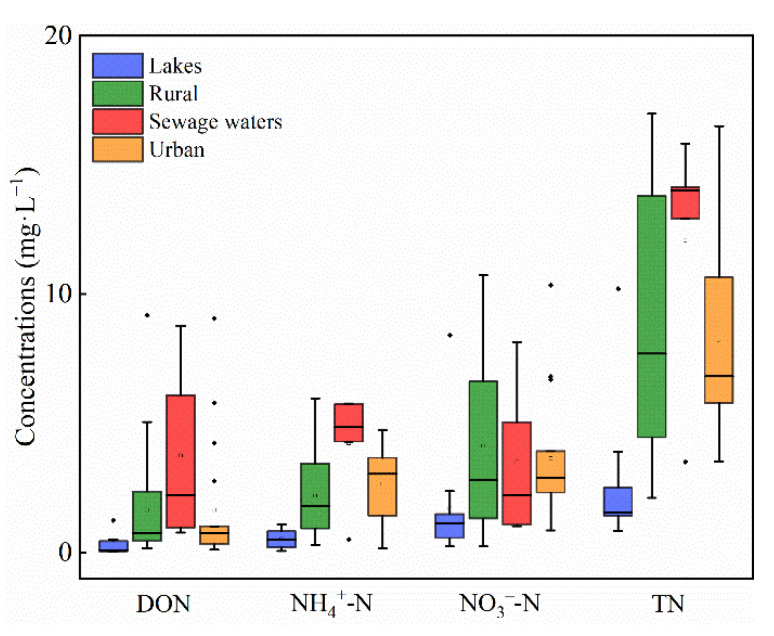
Concentrations of nitrogen species in river waters in Tianjin.

**Figure 4:**
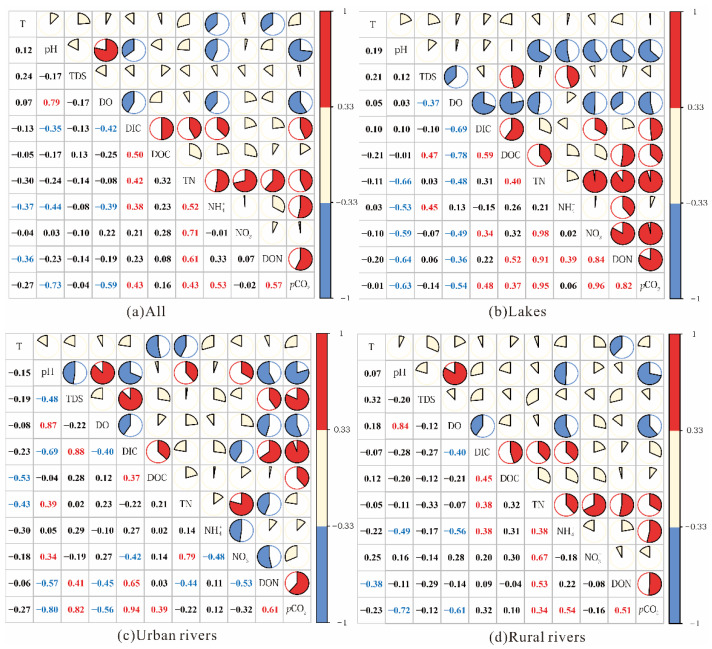
Correlations of water environmental parameters in surface water in Tianjin, China. The colors represent the range of correlation coefficient. Red indicates positive correlations (ranging from 0.33 to 1), blue indicates negative correlations (ranging from −1 to −0.33), and light yellow indicates weak correlations (ranging from −0.33 to 0.33). The ratios of colored areas in circles represent the degree of correlations.

A box plot of nitrogen species in different waters is presented in Figure 3. The concentrations of TN ranged from 0.83 to 10.20 mg·L^−1^ in lakes, 2.11 to 16.97 mg·L^−1^ in rural rivers, 3.51 to 16.51 mg·L^−1^ in urban rivers, and 3.51 to 15.82 mg·L^−1^ in sewage waters, and mean values of NO_3_^−^-N were higher than other nitrogen species (NH_4_^+^-N, NO_2_^−^-N, and DON) except those in sewage-draining waters (Table 2). Rural rivers and sewage-draining waters presented higher nitrogen concentrations in diverse waters, and urban rivers exhibited considerably higher nitrogen concentration than lakes (*p* < 0.05) (Figure 3). Higher NH_4_^+^-N concentrations were observed in anoxic surface waters, such as R24 in urban rivers, R9 and R20 in sewage-draining waters, where the DO concentrations were all below 3.67 mg·L^−1^. Similar findings have been reported from studies in a coastal lagoon [21] and anoxic water layers in the hypolimnion of a lake/reservoir [27] as well. Except the lake waters, the NH_4_^+^-N in surface waters had a negative relationship with concentrations of DO (Figure 4).

DIC ranged from 0.80 mol·L^−1^ to 11.20 mol·L^−1^, with mean values of 3.69, 4.18, 4.52, and 5.41 mol·L^−1^ in lakes, urban rivers, rural rivers, and sewage waters, respectively. DIC in river waters in Tianjin exhibited negative correlations with DO while it was positively related to DOC (Figure 4). The correlations between DIC and each nitrogen species displayed obviously differences (*p* < 0.05), exhibiting positive correlations with NH_4_^+^-N (R = 0.38, *p* < 0.01) and TN (R = 0.42, *p* < 0.01) (Figure 4a). DOC positively correlated to DON in lakes (R = 0.52, *p* < 0.01), with the highest value in the Yongdingxin River (R13, 19.54 mg·L^−1^) and the lowest value in the Yuqiao Reservoir (L1, 1.49 mg·L^−1^).

### 3.3. Partial Pressure of CO_2_ in Different River Waters

In total, the *p*CO_2_ averaged 2663.21 μatm in surface waters in the river networks in Tianjin, fluctuating within a wide range (standard deviation: 4124.99 μatm). Compared with the average values of global rivers (averaging 3100 μatm) [5,14], the *p*CO_2_ in most of the rivers in Tianjin presented a high level. Specifically, the mean values of *p*CO_2_ in sewage waters (11,350.59 ± 7243.23 μatm) were significantly higher than those of urban rivers (3035.41 ± 3510.66 μatm), rural rivers (1516.58 ± 1598.54 μatm), and lakes (628.16 ± 807.27 μatm). The results of Pearson’s correlation coefficient showed that *p*CO_2_ consistently exhibited negative correlations and was statistically significant with pH and DO, while it showed positive correlations with DON (Figure 4). Additionally, *p*CO_2_ was positively related to DIC in river waters in Tianjin, especially in urban rivers, with the highest correlation coefficient (R = 0.94, *p* < 0.01). The significant correlations between nitrogen species (TN, NH_4_^+^-N, NO_3_^−^-N, and DON) and *p*CO_2_ have been observed in lakes and rural rivers.

### 3.4. Fluxes of CO_2_ in River Networks

**Figure 5:**
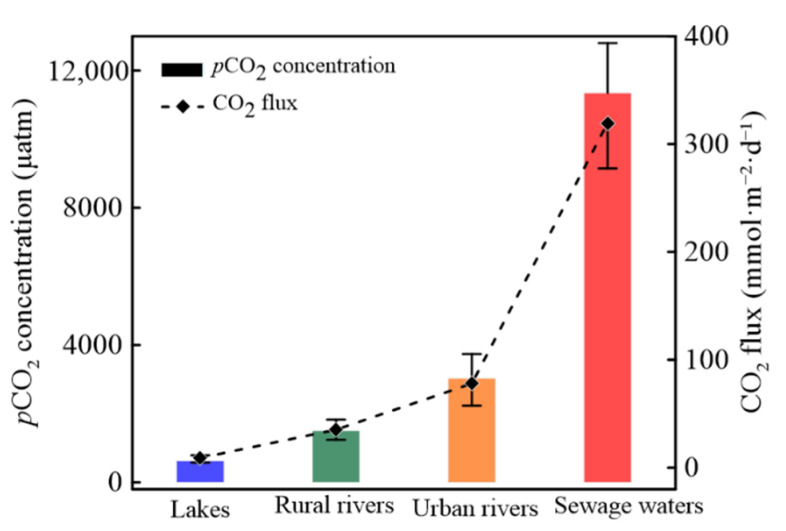
The relationship of *p*CO_2_ concentration and CO_2_ fluxes in river waters in Tianjin.

**Figure 6:**
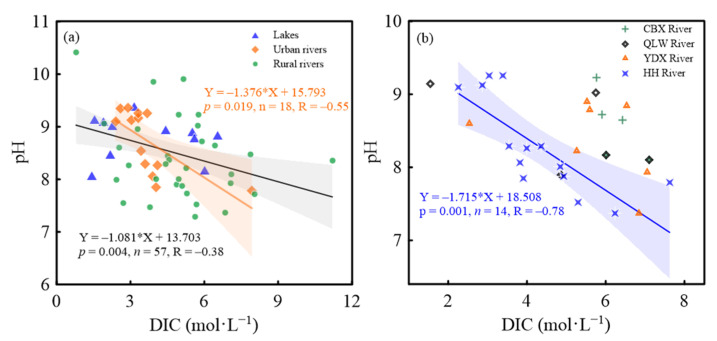
Correlations between DIC and pH in diverse river waters in Tianjin. (**a**) All the river waters; (**b**) Chaobaixin (CBX) River, Qinglongwan (QLW) River, Yongdingxin (YDX) River, and Haihe (HH) River.

Distributions of CO_2_ fluxes were consistent to those of *p*CO_2_, while there were obvious discrepancies when evaluating the various surface waters in Tianjin (Figure 5). Calculated CO_2_ fluxes ranged from −10.47 mmol·m^−2^·d^−1^ (in Ziya River, R23) to 538.93 mmol·m^−2^·d^−1^ (in Beijing-drainage River, R20). The highest CO_2_ fluxes were found in sewage waters (319.54 ± 210.54 mmol·m^−2^·d^−1^) compared to those in urban rivers (77.95 ± 102.30 mmol·m^−2^·d^−1^), lakes (7.75 ± 23.51 mmol·m^−2^·d^−1^), and rural rivers (33.42 ± 46.55 mmol·m^−2^·d^−1^) (Figure 6). Statistically significant differences in CO_2_ fluxes in lakes from those in other river waters (*p* < 0.05) have been observed, while there were no obvious variations between sewage waters and urban rivers (*p* > 0.05). In hypoxic surface waters with higher CO_2_ supersaturated conditions, increased CO_2_ fluxes were also detected, such as the sampling sites L5 in Qilihai Wetland, R35 in Yongdingxin River, and R20 in Beijing-drainage River.

## 4. Discussion

### 4.1. Sewage-Draining Dominated the Spatial Distributions of pCO_2_ in River Networks

Sewage-draining was linked to the aquatic environmental conditions, carbon or nitrogen accumulation, and organic matter decomposition rate, which obviously modified the intensity of riverine contamination levels and CO_2_ emissions [28,32]. Diverse river waters in Tianjin showed different contamination levels, mainly due to the complicated source of sewage-draining waters under intense anthropogenic activities [22,33]. Lakes/reservoirs generally served as the water sources and nature reserves in Tianjin, were less affected by sewage, and expressed the lowest *p*CO_2_ values in Tianjin River networks (Figure 5). Rural rivers generally accepting a large amount of domestic/industrial sewage have overwhelmingly altered aquatic ecosystems, while urban rivers could be considered as landscape waters with drastic disturbance by human activities. Higher *p*CO_2_ values were generally accompanied with the low pH and high DIC in direct sewage-draining waters [11,25]. In this study, sampling sites in sewage-draining waters in Longfeng River (R8 and R9), Beijing-drainage River (R20), and Beitang-drainage River (R45) all presented higher *p*CO_2_ (approximately 4 times that of averaged *p*CO_2_ in worldwide rivers, i.e., 3100 μatm) [5] with lower pH (ranged from 7.29 to 7.85) and higher DIC (ranged from 4.05 to 6.85 mol·L^−^^1^) when compared with other waters in this study.

Traditionally, increased *p*CO_2_ values were consistent with population density [34], and it was applicable in this study (with lower *p*CO_2_ levels in rural rivers and lakes than in urban rivers and sewage waters). Here, we selected the Haihe River to disclose the effects of different pollution sources on *p*CO_2_ in river waters, with the most obvious variations in environmental conditions (Figure 2). The higher *p*CO_2_ values exhibited in R8 and R20 along the Haihe River were mainly attributed to the direct reception of large amounts of sewage in the upper reaches [11]. A similar observation was also made in urban rivers with direct sewage-draining waters in Beitang-drainage River (R45). However, compared with those in rural rivers, lower *p*CO_2_ values in urban areas (especially in R35, R36, and R37) might result from the artificial dredging project which reduced biological respiration and organic matter degradation in river waters and sediments [35]. Additionally, the positive correlations between *p*CO_2_ and DOC/DON in urban rivers (Figure 4) also indicated that increased organic matter played important roles in the CO_2_ production in urban rivers. These results highlighted that sewage-draining would explain the differences in environmental conditions and spatial variations in *p*CO_2_ in river waters, and sewage inputs increased *p*CO_2_ levels.

### 4.2. Biogeochemical Processes Served as the Primary Mechanisms of CO_2_ Production

It has been noticed that there are two production mechanisms of CO_2_ in waters: the chemical weathering and the biological processes. Chemical weathering dissolves silicate or carbonate rocks by consuming CO_2_ and generating DIC, which obviously occurred in the Karst watershed in China [9,24,36]. Biologically, the photosynthesis and respiration of aquatic plants, microbial respiration and reproduction, and organic matter degradation commonly dominate CO_2_ production and release [13]. Additionally, eutrophic waters in Tianjin provided favorable conditions for phytoplankton reproduction, and high temperatures in the rainy season also stimulated microbial activities, which traditionally promote the primary productivity of phytoplankton fixing a large amount of CO_2_ as important carbon sinks in the water [22,37].

Generally, water–rock reaction, CO_2_ input from the soil aeration zone, and the dissolution of atmospheric CO_2_ are commonly regarded as the primary sources of CO_2_ in rivers [17,24,28]. However, because of low cover rates of exposed rocks or outcrops in the studied river watersheds, and the geological type was mainly quaternary sediment, and the water–rock reaction in the studied river waters could be neglected. Generally, the correlations of pH and DIC have been used to characterize the influencing factors of CO_2_ production and transformations in river waters [21]. In this study, pH presented negative correlations with DIC in all the river waters (including lake waters and river waters) (R = −0.38, *p* < 0.01) including Haihe River (R = −0.78, *p* < 0.01) (Figure 6), revealing the potential contributions of photosynthetic processes to DIC uptake and CO_2_ consumption in water, but they have obviously been affected by other processes and influencing factors [13]. Specifically, most of the urban rivers and lakes have very low water flow and long residence time, providing ideal static conditions for biogeochemical carbon transformations, including photosynthesis, respiration, and organic matter degradation [25]. On the one hand, in rural rivers and sewage rivers, frequent runoff processes in the rainy season during the sampling period transferred CO_2_ from the soil aeration zone to river water, increasing the *p*CO_2_ levels in the rural river water; on the other hand, a large amount of organic carbon/nitrogen input from drainage waters would promote CO_2_ production by stimulating the organic matter degradation and biological respiration in sewage waters. Additionally, in the lakes and urban rivers, good positive relationships between DOC and *p*CO_2_ (Figure 4) indicated that CO_2_ production was mainly dominated by internal organic matter degradation and biological respiration.

Even though most of the river waters in the study area were sources of atmospheric CO_2_ in the rainy season, unsaturated *p*CO_2_ has been found in some of the sites in the lakes (L2, L4, L9, and L10) and urban areas (R33, R34, R37, R38, R39, R42, and R43), which may be attributed to the photosynthesis enhanced by algal bloom and well-growing aquatic plants in the rainy season with proper temperature and nutrient supply [20,33,38].

### 4.3. Effects of Carbon/Nitrogen Increases on pCO_2_

Dramatic anthropogenic activities with increasing pollutant discharge have contributed to complicated environmental conditions in river ecosystems, and potentially provided huge challenges to biochemical processes [15,18]. In lakes, positive correlations of DON, inorganic nitrogen (NO_3_^−^-N and NH_4_^+^-N), and DOC have been observed, especially the significantly positive correlations between NO_3_^−^-N and DON (R = 0.84, *p* < 0.01) (Figure 4). Generally, higher temperatures and carbon/nitrogen inputs relatively encouraged microbial activities and influenced the contributions of *p*CO_2_ in river ecosystems [8,12,25]. Nutrient supplementation was also verified as an important factor influencing *p*CO_2_ by enhancing the primary production and mineralization of organic matter [39,40,41]. Therefore, CO_2_ emissions of lakes were mainly controlled by photosynthesis and mineralization of organic matter, which could be disclosed by the correlations between environmental parameters and nitrogen species.

Higher concentrations of NH_4_^+^-N have been observed in the Longfeng River (R8 and R9), Beijing-drainage River (R20), and Jiyun River (R17), which correspond to the amount of sewage waters produced by anthropogenic activities. The concentrations of NH_4_^+^-N are normally considered as the crucial indicator of sewage discharges from anthropogenic activities [42], which have been found to be the highest in sewage waters (Table 2). Consistently with previous studies, increased *p*CO_2_ with higher nitrogen concentrations was also found [43]. On the other hand, the positive correlations between NH_4_^+^-N and *p*CO_2_ (R = 0.54, *p* < 0.01) also verified the effect of nitrogen inputs from sewage-draining waters on *p*CO_2_ levels in rural rivers and sewage waters.

**Figure 7:**
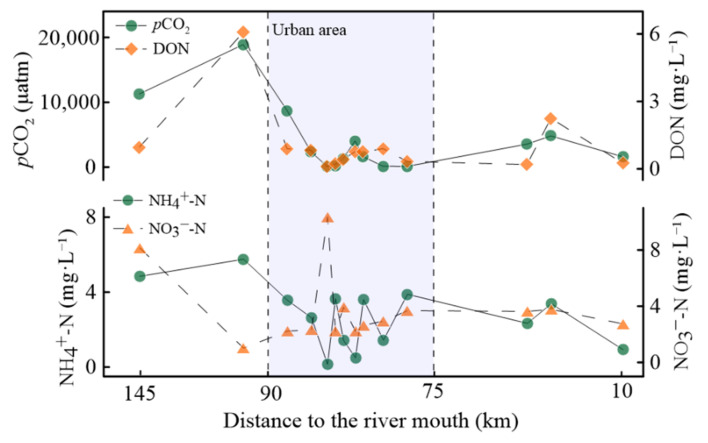
Longitudinal variations in *p*CO_2_ concentrations and nitrogen concentrations along the Haihe River.

The distributions of nitrogen species (NH_4_^+^-N, NO_3_^−^-N, and DON), environmental parameters, and *p*CO_2_ along the Haihe River, with distinct longitudinal variations (Figure 2 and Figure 7), would be effective evidence on the influence of carbon/nitrogen concentrations. Urban rivers impacted more by anthropogenic activities undergoing intense biochemical processes exhibited higher *p*CO_2_ levels [22,36]. The concentrations of NH_4_^+^-N were roughly decreased while DO and pH levels increased, in contrast to NO_3_^−^-N (especially in urban areas), demonstrating that the CO_2_ emissions from river waters were produced by the microbial degradation under aerobic and hypoxic conditions [44]. The highest NO_3_^−^-N (10.35 mg·L^−1^) and DOC (9.07 mg·L^−1^) but lower NH_4_^+^-N (0.15 mg·L^−1^) and DON (0.10 mg·L^−1^) in R37 have been observed along the Haihe River, which could be explained by the principial biogeochemical processes in this river water being aerobic mineralization (DO saturation was 131%). Obviously positive correlations along the Haihe River between *p*CO_2_ and DON (R = 0.84, *p* < 0.01) and DIC (R = 0.78, *p* < 0.01) illustrated that higher nutrient concentrations stimulated microbial activities, and the reception of wastewaters enhanced *p*CO_2_ values [33,45]. Thus, CO_2_ emissions should be investigated in detail because of the complexity of sources of carbon/nitrogen concentrations.

### 4.4. CO_2_ Fluxes

Similar to most river ecosystems under intense anthropogenic activities receiving a substantial amount of nutrients, CO_2_ fluxes demonstrated obvious positive correlations with the *p*CO_2_ values in Tianjin (*n* = 57, *p* < 0.01). In general, CO_2_ emissions from river ecosystems were mainly dominated by *p*CO_2_ levels at the air–water interface and gas transfer velocity [4,5,14]. As shown above, variations in averaged CO_2_ fluxes in river waters were higher in sewage waters and urban rivers while lower in lakes and rural rivers, illustrating the stronger effects of carbon/nitrogen concentrations on CO_2_ emissions in this study. CO_2_ fluxes from other river waters (rural rivers, urban rivers, and lakes) around the world are exhibited in Table 3. CO_2_ fluxes in urban rivers in Tianjin were estimated to be marginally lower than those in highly polluted eutrophic rivers [25,46], while significantly higher than those in other rural rivers [9,25,47]. CO_2_ fluxes in sewage waters exhibited extremely high levels compared with those of natural rivers. The annual CO_2_ fluxes were estimated to be 1.08 × 10^13^ mmol (i.e., 1.08 × 10^–4^ Pg C) according to the water area of the Tianjin River network (with 884 km^2^) [22], which was a major source of atmospheric CO_2_. Despite the fact that projected values of CO_2_ emissions in this study were lower than global CO_2_ emissions from river ecosystems (1.80 Pg C·yr^−1^ from rivers and 0.32 Pg C·yr^−1^ from lakes) [5,6], the vast water area around the world should be given more consideration when considering the effects of CO_2_ emissions from water.

## 5. Conclusions

The study revealed the impacts of urbanization and nitrogen pollution on CO_2_ emissions and production in river networks in Tianjin. Increasing sewage effluent and pollutant discharge in river ecosystems dramatically altered the environmental conditions, potentially triggering biochemical processes and CO_2_ production. The spatial distributions of CO_2_ emissions presented obvious differences mainly attributed to the complexity of sources of sewage-draining waters altering biological processes in river waters. Urban rivers and sewage waters exhibited higher *p*CO_2_ and CO_2_ fluxes than rural rivers and lakes, which might be dominated by the enhanced biochemical processes of respiration with higher carbon/nitrogen concentrations. Sampling sites with unsaturated *p*CO_2_ levels in lakes and urban rivers were mainly controlled by photosynthesis and organic degradation. The correlations between *p*CO_2_ and organic carbon/nitrogen demonstrated that nutrient accumulation stimulated microbial activities, and the reception of wastewaters could enhance *p*CO_2_ levels. We highlighted the correlations between *p*CO_2_ and carbon/nitrogen species in a regional river network and provided a theoretical basis for water management when considering CO_2_ emissions under dramatic anthropogenic activities.

## Figures and Tables

**Table 1:** Statistics of the environmental variables in surface water in Tianjin, China.

Types	T (ºC)	pH	TDS (mg·L^−1^)	EC (μs·cm^−1^)	DO (mg·L^−1^)	Alk (mg·L^−1^)
Lakes	27.50–34.80	8.07–9.39	250.00–20,100.00	501.00–40,900.00	6.20–8.93	87.19–418.03
(*n* = 11)	30.33 ± 2.04	8.80 ± 0.14	2847.09 ± 5788.55	5775.00 ± 11,784.55	7.60 ± 0.83	237.23 ± 122.84
Rural rivers	25.80–30.90	7.88–10.41	269.00–22,900.00	537.00–45,800.00	4.48–14.24	101.38–689.06
(*n* = 23)	28.50 ± 1.64	8.69 ± 0.68	4183.68 ± 6159.65	8065 ± 12,066.92	8.43 ± 2.90	314.01 ± 1315.01
Urban rivers	25.60–30.40	7.47–9.36	484.00–32,800.00	970.00–6570.00	2.15–12.58	155.45–471.43
(*n* = 18)	28.30 ± 1.63	8.44 ± 0.70	1123.83 ± 692.65	2250.28 ± 1386.33	8.24 ± 2.49	258.84 ± 98.01
Sewage	26.70–29.60	7.29–7.90	418.00–902.00	834.00–1802.00	3.32–6.84	240.95–381.20
(*n* = 5)	27.58 ± 1.16	7.59 ± 0.28	635.40 ± 232.78	1269.40 ± 466.70	4.92 ± 1.61	308.94 ± 51.04

Note: The data shown above are minimum–maximum, and average values ± mean deviation.

**Table 2:** Statistics of the nitrogen species in surface water in Tianjin, China.

Types	TN (mg·L^−1^)	NH_4_^+^-N (mg·L^−1^)	NO_3_^−^-N (mg·L^−1^)	NO_2_^−^-N (mg·L^−1^)	DON (mg·L^−1^)
Lakes	0.83–10.20	0.06–1.08	0.23–8.41	0.01–0.29	0.03–1.24
(*n* = 11)	2.57 ± 2.66	0.52 ± 0.35	1.69 ± 2.31	0.07 ± 0.10	0.29 ± 0.36
Rural rivers	2.11–16.97	0.30–5.96	0.23–10.72	0.01–4.90	0.16–9.17
(*n* = 23)	8.71 ± 4.93	2.18 ± 1.62	4.12 ± 3.27	0.83 ± 1.42	1.64 ± 2.06
Urban rivers	3.51–16.51	0.15–4.73	0.85–10.35	0.01–1.65	0.10–9.05
(*n* = 18)	8.08 ± 3.52	2.65 ± 1.43	3.64 ± 2.23	0.16 ± 0.39	1.64 ± 2.40
Sewage	3.51–15.82	0.50–5.76	1.02–8.15	0.02–2.83	0.77–8.77
(*n* = 5)	12.08 ± 4.90	4.23 ± 2.17	3.50 ± 3.07	0.60 ± 1.25	3.76 ± 3.52

Note: The data shown above are average values ± mean deviation. PON is particle organic nitrogen.

**Table 3:** Fluxes of CO_2_ in other rivers/lakes.

	Name	Location	CO_2_ Fluxes (mmol·m^−2^·d^−1^)	References
Urban rivers	Jacarepagua Lagoon Complex	Southeastern Brazil	22~48	[21]
	Hou River	China	403 ± 204	[25]
	Red River	Vietnam	503 ± 16.9	[46]
	Hooghly River	India	2.64~12.00	[11]
	Tianjin river network	China	77.95 ± 102.30	This study
Rural rivers	Nome Creek	Alaska	5.21 ± 3.94	[48]
	Ob River	Western Siberia	–1.67~745	[47]
	Wujiang River	China	176~239.7	[9]
	Chaoyang River	China	116 ± 88	[25]
	Tianjin River network	China	35.33 ± 46.94	This study
Lakes	Jacarepagua Lagoon	Southeastern Brazil	4.27~18.99	[21]
	Eastmain Reservoir	Canada	53.4~158.6	[49]
	Dongfeng Lake	China	13.4	[9]
	Tianjin River network	China	7.75 ± 22.42	This study

## Data Availability

The datasets used or analyzed during the current study are available from the corresponding author on reasonable request.
